# A retrospective analysis of factors associated with the length of hospital stay in COVID-19 patients treated with Nirmatrelvir / Ritonavir

**DOI:** 10.3389/fphar.2023.1146938

**Published:** 2023-06-05

**Authors:** Jiantao Zheng, Wencong Hong, Chanjuan Zhou, Donghuang Hong, Hong Yan, Yanghui Shen

**Affiliations:** ^1^ Department of Emergency, Quanzhou First Hospital Affiliated to Fujian Medical University, Quanzhou, China; ^2^ Department of Respiratory and Critical Care Medicine, The Hospital of Nanan City, Nanan, China; ^3^ Department of Geriatrics, Quanzhou First Hospital Affiliated to Fujian Medical University, Fuzhou, China; ^4^ Department of Critical Care Medicine, Shengli Clinical Medical College of Fujian Medical University, Fuzhou, China; ^5^ Department of Critical Care Medicine, Fujian Provincial Hospital, Fuzhou, China; ^6^ The First School of Clinical Medicine, Southern Medical University, Guangzhou, China

**Keywords:** coronavirus disease 2019 (COVID-19), length of hospital stay, Nirmatrelvir/Ritonavir, Omicron BA.2, retrospective analysis

## Abstract

**Objectives:** This study reviewed factors influencing the length of hospital stay in adult inpatients with confirmed Coronavirus disease (COVID-19) who were treated with Nirmatrelvir/Ritonavir.

**Methods:** We did a retrospective analysis of data from a cohort of inpatients with confirmed diagnosis of Omicron variant of SARS-CoV-2 infection who were treated with Nirmatrelvir/Ritonavir. We included patients who were treated from 13th March 2022 to 6th May 2022 in various in-patient treatment units in Quanzhou, Fujian Province, China. The primary study outcome was the length of hospital stay. Secondary study outcome was viral elimination defined as negative for ORF1ab and N genes [cycle threshold (Ct) value ≥35 in real-time PCR], according to local guidelines. Hazard ratios (HR) of event outcomes were analyzed using Multivariate Cox regression models.

**Results:** We studied 31 inpatients with high risk for severe COVID-19 who were treated with Nirmatrelvir/Ritonavir. We found that inpatients with shorter length of hospital stay (≤17 days) were mostly females with lower body mass index (BMI) and Charlson Comorbidity Index (CCI) index. Their treatment regimen with Nirmatrelvir/Ritonavir was started within 5 days of diagnosis (*p* < 0.05). Multivariate Cox regression indicated that inpatients starting treatment of Nirmatrelvir/Ritonavir within 5 days had a shorter length of hospital stay (HR 3.573, *p* = 0.004) and had a faster clearance of viral load (HR 2.755, *p* = 0.043).

**Conclusion:** This study assumes relevance during the Omicron BA.2 epidemic as our findings suggest that early treatment with Nirmatrelvir/Ritonavir within 5 days of diagnosis (≤5 days) was highly effective in shortening the length of hospital stay and faster viral load clearance.

## 1 Introduction

The Coronavirus disease 2019 (COVID-19) epidemic, caused by the severe acute respiratory syndrome coronavirus 2 (SARS-CoV-2), has spread worldwide and emerged as a serious public health problem. More than 500 million confirmed cases and 6.3 million deaths were reported worldwide as of 15th May 2022 (Worldometer, 2022). The Omicron BA.2 variant has higher infectivity, stronger vaccine breakthrough ability, and higher antibody drug resistance (Walls et al., 2020; Dejnirattisai et al., 2022a; Dejnirattisai et al., 2022b; Kawaoka et al., 2022; Liu et al., 2022; Meng et al., 2022; Migueres et al., 2022; Mohapatra et al., 2022).

Earlier studies have reported treating COVID-19 with multiple antiviral agents, monoclonal antibodies, and immunomodulators, but none of these treatments have shown significant clinical efficacy for BA.2 strain (Drożdżal et al., 2021; Weinreich et al., 2021; Ohashi et al., 2022; VanBlargan et al., 2022). Nirmatrelvir/Ritonavir targets 3CLpro inhibitor (Nirmatrelvir) and cytochrome CYP3A4, thereby increasing Nirmatrelvir serum levels (ritonavir) (Hammond et al., 2022). Results from the Phase III clinical trial of Nirmatrelvir/Ritonavir (EPIC-HR) showed reduced risk of hospitalization and mortality caused by the Delta variant of COVID-19 in outpatients with high risk. There is limited evidence of Nirmatrelvir/Ritonavir’s efficacy on Omicron variant infections in high risk inpatients. On 11th February 2022, the National Medical Products Administration (NMPA) of China approved Nirmatrelvir/Ritonavir as the standard treatment for adult patients with mild to moderate COVID-19, who had risk factors for progression to severe disease. The first case of Omicron BA.2 variant of COVID-19 was diagnosed in Quanzhou City, China on 13th March 2022. Since then, 32 inpatients with high viral load were treated with Nirmatrelvir/Ritonavir.

Hence, we decided to conduct a small retrospective cohort study of COVID-19 inpatients to verify the effectiveness of Nirmatrelvir/Ritonavir in shortening length of hospital stay and hastening clearance of viral load.

## 2 Materials and methods

### 2.1 Study subjects

The study subjects were adult inpatients with confirmed Omicron BA.2 variant of COVID-19 who were admitted in makeshift/mobile field hospitals, isolation wards, and intensive care units in Quanzhou City, Fujian Province. The study period was from 13th March 2022 to 6th May 2022. This study was reviewed and approved by the Ethics Committee of Quanzhou 1st Hospital Affiliated to the Fujian Medical University [Quan Yilun (2022) 162].

### 2.2 Criteria for diagnosis

According to the National Health Commission of the People’s Republic of China diagnosis and treatment protocol for COVID-19 (trial version 9) (National Health Commission of the People’s Republic of China, 2022), there are four groups of clinical classification of cases: mild, moderate, severe, and critical. The details are as follows:

Mild: Mild clinical symptoms, no pneumonia manifested in imaging.

Common type: With the above clinical manifestations, pneumonia can be seen on imaging.

Severe: 1) Shortness of breath, RR ≥ 30 times/min; 2) In a resting state, oxygen saturation ≤ when inhaling air 93%; 3) Arterial blood oxygen partial pressure (PaO2)/inhaled oxygen concentration (FiO2) ≤ 300 mmHg (1 mmHg = 0.133 kPa), high altitude (over 1,000 m above sea level) areas should correct PaO2/FiO2 according to the following formula: PaO2/FiO2 × [760/atmospheric pressure (mmHg)]; 4) The clinical symptoms are progressively aggravated, and lung imaging shows that the lesion progresses >50% within 24–48 h.

Critical: 1) Respiratory failure occurs and requires mechanical ventilation; 2) Shock occurs; 3) Combining with other organ failure requires ICU monitoring and treatment.

### 2.3 Criteria for admission and discharge, principles of respiratory support treatment

Admission criteria were as follows: Once the patient is diagnosed, he should be transferred immediately (within 2 h) to a designated hospital for treatment or release the cabin hospital for centralized isolation and treatment, so all confirmed patients are admitted to the hospital. Discharge criteria were as follows: 1) Normal body temperature for more than 3 days; 2) Significantly recovered respiratory symptoms; 3) Lung CT imaging shows obvious absorption and recovery; 4) Ct value ≥35 of N gene and ORF gene as detected by real-time PCR of COVID-19 (National Health Commission of the People’s Republic of China, 2022). The principles of respiratory support treatment were as follows: 1) Severe patients diagnosed as PaO2/FiO2 lower than 300 mmHg are given oxygen therapy; 2) PaO2/FiO2 lower than 200 mmHg are given nasal high-flow oxygen therapy (HFNC) or non-invasive ventilation (NIV); 3) PaO2/FiO2 If FiO2 is lower than 150 mmHg, especially for patients with significantly increased inspiratory effort, endotracheal intubation and invasive mechanical ventilation should be performed.

### 2.4 Inclusion and exclusion criteria

Inclusion criteria: 1) Patients who fulfilled the diagnostic criteria; 2) Age ≥18 years; 3) Treated with a full course of Nirmatrelvir/Ritonavir antiviral therapy during hospitalization as follows: Patients with normal renal function and mild renal dysfunction [estimated glomerular filtration rate (eGFR) 60–90 mL/min/1,73 m^2^ (Kawaoka et al., 2022)] were administered 300 mg Nirmatrelvir (150 mg/tablet) and 100 mg Ritonavir (100 mg/tablet). Three tablets were orally administered twice daily for 5 days. In patients with moderate renal impairment [estimated e GFR30 ∼ 60 mL/min/1,73 m^2^ (Kawaoka et al., 2022)], the dosage was adjusted to 150 mg Nirmatrelvir and 100 mg Ritonavir were orally administered twice daily for 5 days.

We excluded patients who were younger than 18 years, those who were pregnant, had contraindications, and those who did not complete the oral 5-day course of Nirmatrelvir/Ritonavir.

### 2.5 Data collection

We used the Haitai Medical Records System (Nanjing Haitai Medical Information System Co., Ltd.) to collect clinical data. Details collected included gender, age, BMI, underlying diseases, complications, smoking history, number of vaccinations, disease classification, type of hospitalization, basic parameters at the time of admission (whole blood lymphocyte count, plasma Interleukin 6 (IL-6), plasma C-reactive protein (CRP)), the time of initiating Nirmatrelvir/Ritonavir treatment, Ct value before treatment, liver and kidney functions before and after treatment, coagulation function before and after treatment, routine blood tests results before and after treatment, the time taken to reach Ct value ≥35 of N gene and ORF gene, and the length of hospital stay.

### 2.6 Indicators and definitions

#### 2.6.1 Outcome

The primary outcome was defined as the length of the hospital (compared with a median number of days in the cohort), and the secondary outcome was the time taken to reach a Ct value ≥35 of the N gene and ORF gene. The survival time is the time from the patient’s admission to the occurrence of the positive event. In this study, positive events were defined as the primary outcome, “patient discharged from hospital,” and the secondary outcome, “nucleic acid CT value > 35”. Therefore, the follow-up duration was defined as the time from the patient’s admission to discharge from the hospital. Moreover, all 31 patients were discharged cured after the diagnosis of novel coronavirus infection was confirmed and admitted within 24 h, and all met the discharge criteria of the cited “Novel Coronavirus Pneumonia Treatment Protocol (Trial Version 9)", with no censoring events and a 100% positive event rate.

#### 2.6.2 Influencing factors

We analyzed the following variables.

1) Underlying diseases, which included cardiovascular and cerebrovascular diseases, chronic lung diseases, diabetes, chronic liver and kidney diseases, tumors, immunodeficiency, and so on.
2) Complications, which included gastrointestinal bleeding, infection with other pathogens, abnormal liver function, coagulation dysfunction, allergic dermatitis, and so on.
3) Charlson Comorbidity Index (CCI), which provides an integrated evaluation of the patient’s comorbidities (Charlson et al., 1994).
4) Risk classification criteria of inpatients.
5) The Ct value before Nirmatrelvir/Ritonavir was administered.
6) The time taken to reach of Ct value ≥35.
7) Time of administering Nirmatrelvir/Ritonavir

### 2.7 Statistical analyses

We used SPSS 25.0 statistical software (IBM, New York) for the statistical analysis of data. The Kolmogorov-Smirnov test was used to assess the normality of the distribution of continuous variables. Continuous variables measurement conforming to the normal distribution were expressed as mean ± standard deviation (–x ± *s*), or as median (interquartile range) [M (Q1, Q3)]. Categorical variables were presented as frequencies (percentages). Independent sample t-test, Wilcoxon rank-sum test, Fisher’s exact probability test, and the Chi-square test were used for comparing the two groups (the short hospitalization group and the long hospitalization group). Schoenfeld residuals method was used to examine whether each factor met the PH hypothesis test. Univariate Cox regression analysis was used to estimate the hazard ratios (HRs) and 95% confidence interval (CI) of the effect of each variable on the outcome for factors included in the proportional hazards (PH) assumption. Log-rank test was used to compare the survival distribution. Tests of significance were done using R version 4.2.0 (University of Auckland, Auckland) and the Survminer R package was used to plot the survival curve. We used Multivariate Cox regression models to analyze HRs of event outcomes. *p* < 0.05 was considered statistically significant.

## 3 Results

### 3.1 General information

Initially, 32 inpatients were included in the study. Of them, one patient did not complete the 5-day Nirmatrelvir/Ritonavir treatment due to personal reasons. The final cohort consisted of 31 inpatients whose data we collected. There were 17 males and 14 females, with a median age of 42 years, median time of reaching Ct value ≥35 was 12 days, and median length of hospital stay was 17 days.

Sixteen inpatients belonged to the short hospitalization group (≤17 d) and 15 inpatients belonged to the long hospitalization group (>17 d).

#### 3.1.1 Population characteristics

There were no statistical differences in age, gender composition ratio, completion rate of 3 doses of vaccine, BMI >25 kg/m^2^, laboratory tests (lymphocyte count, IL-6, CRP), risk classification criteria, and nucleic acid CT value before treatment between the two groups (*p* > 0.05). There was no significant difference in the underlying diseases and complications between the two groups (*p* > 0.05), but there was a significant difference in the Charlson comorbidity index (CCI) ≥ 1 between the two groups (*p* = 0.043). Population characteristics and group comparisons are detailed in Table 1.

**TABLE 1:** Clinical characteristics and group comparison.

Variable factors	Total (*n* = 31)	Short hospitalization group (*n* = 16)	Long hospitalization group (*n* = 15)	P
**Gender**				
Female	14 (45.2%)	10 (62.5%)	4 (26.7%)	0.073
Male	17 (54.8%)	6 (37.5%)	11 (73.3%)	
**Age ≥ 60 years, n (%)**	10 (32.3)	4 (25.0)	6 (40.0)	0.458
BMI > 25 kg/m^2^, n (%)	7 (22.6)	6 (37.5)	1 (6.7)	0.083
**Smoking**	2 (6.5%)	1 (6.3%)	1 (6.7%)	>0.99
**Underlying disease** ^a^	14 (45.2%)	5 (31.3%)	9 (60%)	0.156
**Comorbidities** ^b^	9 (29%)	2 (12.5%)	7 (46.7%)	0.054
**Charlson comorbidity index (CCI) ≥ 1, n (%)**	11 (35.5)	2 (12.5)	9 (60.0)	0.009
**Completed 3 doses of vaccine**	14 (45.2%)	8 (50%)	6 (40%)	0.722
**clinical classification**				
mild	18 (58.1%)	10 (62.5%)	8 (53.3%)	0.166
moderate	10 (32.3%)	6 (37.5%)	4 (26.7%)	
severe and critical ill^c^	3 (9.7%)	0	3 (20%)	
**Admission ICU**	4 (12.9%)	0	4 (26.7%)	0.043
**Abnormal laboratory findings on admission**				
Lymphocytes decreased	15 (48.4%)	7 (43.8%)	8 (53.3%)	0.724
IL-6 increased	20 (64.5%)	12 (75%)	8 (53.3%)	0.273
CRP increased	12 (38.7%)	6 (37.5%)	6 (40%)	>0.99
**Other treatments**				
Steroids	2 (6.5%)	2 (13.3%)	0 (0%)	0.226
Anticoagulants	3 (9.7%)	1 (6.3%)	2 (13.3%)	0.6
Antibiotics	4 (12.9%)	0	4 (26.7%)	0.043
**Nirmatrelvir/ritonavir Initiate Time**				
**≤ 5 d**	20 (64.5%)	15 (93.8%)	5 (33.3%)	0.001
**> 5 d**	11 (35.5%)	1 (6.3%)	10 (66.7%)	
**Ct value before administration**				
ORF	22.31 ± 6.08	22.94 ± 6.03	21.66 ± 6.28	0.568
N	22.22 ± 5.98	22.42 ± 6.08	22 ± 6.08	0.85
**Time to resolution (days)**	2 (0.5, 5.5)	2 (1, 4.5)	4 (0, 6.5)	0.535
**The time of Ct values ≥ 35 (days)**	13.74 ± 7 .45	9.63 ± 3.2	18.13 ± 8.24	0.001
**Length of hospital stay (days)**	17 (14, 22.5)	14 (12.5, 14.5)	22.5 (19, 28)	<0.001

^a^ : Short hospitalization group: 4 cases of cardiovascular and cerebrovascular diseases, 1 case of chronic lung diseases, 2 cases of immunodeficiency, and 1 case of diabetes mellitus. Long hospitalization group: 4 cases of cardiovascular and cerebrovascular diseases, 3 cases of chronic lung diseases; 2 cases of chronic kidney diseases; 4 cases of tumors; 4 cases of immunodeficiency; 2 cases of diabetes.

^b^ : Short hospitalization group: 1 case of abnormal liver function, 2 cases of other pathogen infection. Long hospitalization group: 1 case of gastrointestinal bleeding, 4 cases of infection with other pathogens, and 2 cases of coagulation dysfunction.

^c^ : Including 1 case of critical type in the long hospitalization group.

#### 3.1.2 Treatment course

ICU admission rate was higher in the long hospitalization group (4 cases, 26.7%) than the short hospitalization group (0 cases, 0.0%), and the difference was statistically significant (*p* = 0.043).

Nirmatrelvir/Ritonavir initiation time ≤5 days was significantly different in the short hospital group when compared with the long hospital group (93.8% vs. 33.3%, respectively) (*p* = 0.001). The treatment course characteristics and group comparisons are detailed in Table 1.

### 3.2 Primary outcome: length of hospital stay

#### 3.2.1 Univariate analysis

Univariate analysis for factors influencing the PH hypothesis test: male sex (HR 0.358, 95% CI 0.159–0.803, *p* = 0.013) and CCI ≥1 (HR 0.443, 95% CI 0.203–0.965, *p* = 0.040); BMI >25 kg/m^2^ (Kawaoka et al., 2022) (HR 3.819, 95% CI 1.422–10.260, *p* = 0.008), Nirmatrelvir/Ritonavir initiation time ≤ 5 d (HR 4.403, 95% CI 1.842–10.53, *p* = 0.001) (Table 2).

**TABLE 2:** Primary outcome (Length of stay) univariate Cox regression.

Variable factors	HR	95% CI		
		Lower limit	Upper limit	*p*-value
**Gender: Male**	0.358	0.159	0.803	0.013
**Age ≥ 60 years**	0.838	0.39	1.799	0.650
BMI > 25 kg/m^2^	3.819	1.422	10.260	0.008
**Smoking: Yes**	1.980	0.458	8.567	0.361
**Underlying disease: Yes**	0.633	0.304	1.319	0.222
**Comorbidities: Yes**	0.564	0.247	1.283	0.172
**CCI ≥ 1**	0.443	0.203	0.965	0.040
**Vaccination: 3 doses completed**	0.982	0.465	2.075	0.962
**Typing**	0.907	0.555	1.48	0.695
**Laboratory tests on admission**				
Lymphocyte count: decreased	1.325	0.628	2.797	0.460
IL-6: Increased	2.088	0.892	4.887	0.090
CRP: Increased	1.001	0.472	2.122	0.999
**Nirmatrelvir/Ritonavir initiate time (≤ 5d)**	4.403	1.842	10.53	< 0.001
**Ct value before administration**				
ORF	0.972	0.915	1.032	0.351
N	0.953	0.893	1.018	0.151
**Time to resolution (days)**	0.986	0.929	1.047	0.640

**FIGURE 1:**
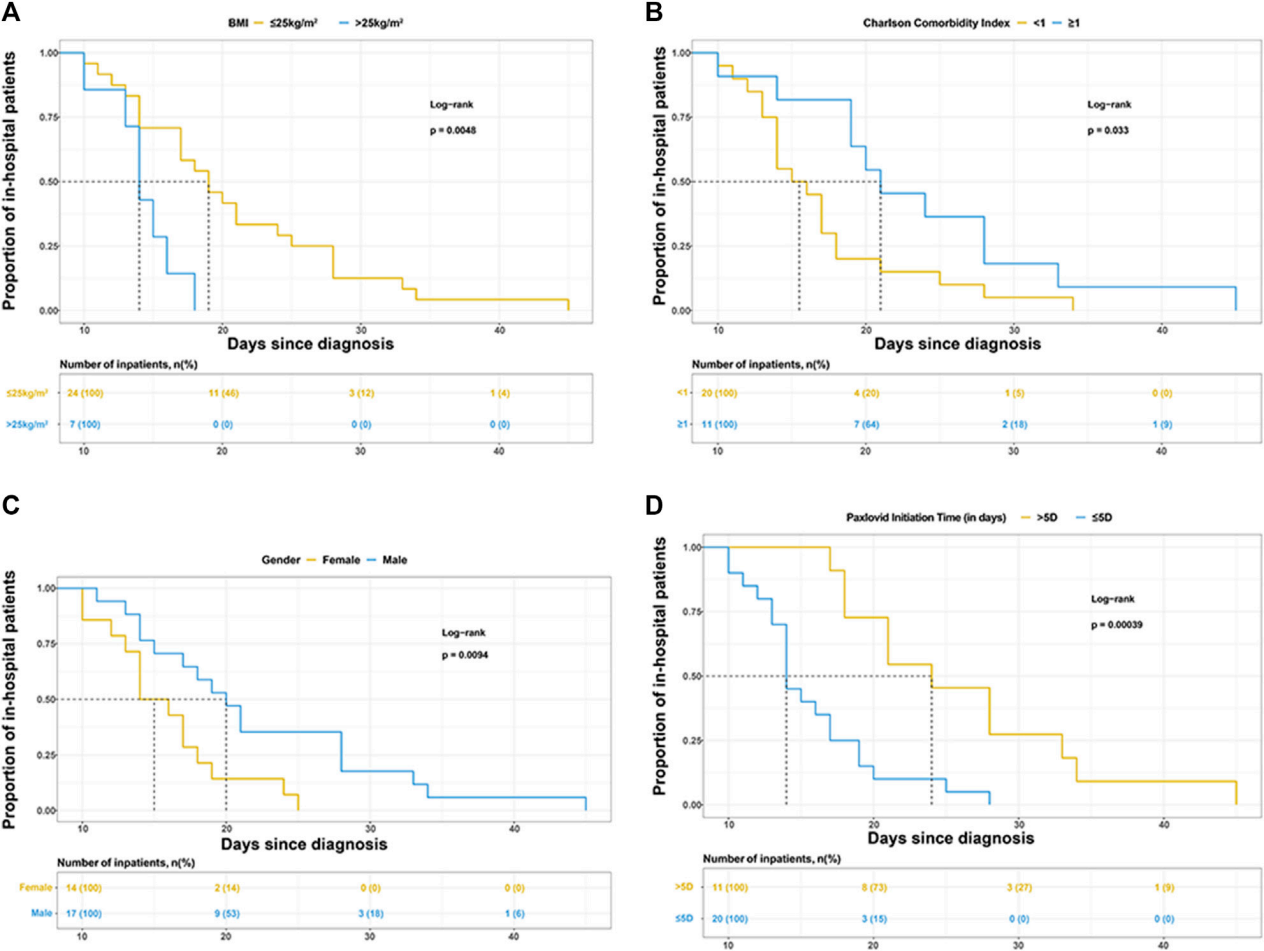
Kaplan-Meier curves for the proportion of in-hospital patients infected with SARS-COV-2 in different groups. The median (interquartile range) length of hospital stay was **(A)** 19 days (14–26.5 days) in patients with BMI ≤25 kg/m2 and 14 days (13–16 days) in patients with BMI>25 kg/m2, **(B)** 15.5 days (13.5–18 days) in patients with Charlson comorbidity index (CCI) < 1 and 21 days (19–28 days) in patients with CCI >1, **(C)** 20 days (15–28 days) in male and 15 days (13–18 days) in female, **(D)** 14 days (13–18 days) in patients treated with Paxlovid within 5 days since diagnosis and 24 days (18–33 days) in patients treated with Paxlovid beyond 5 days since diagnosis.

Gender, BMI, CCI category, and Nirmatrelvir/Ritonavir initiation time were selected to draw the Kaplan-Meier (K-M) survival curves in Figures 1A–D.

#### 3.2.2 Modification of multivariate Cox regression model

Multivariate Cox regression model was performed using independent variables: basic information (gender, BMI, age ≥60 years), underlying disease information (CCI ≥1), treatment and prevention (Nirmatrelvir/Ritonavir initiation time, completion of 3 vaccinations), and disease condition (severity classification). We found that Nirmatrelvir/Ritonavir initiation time ≤5 days was conducive to facilitate patient discharge (HR 3.835, 95% CI 1.330–11.062, *p* = 0 .013), as shown in Table 3.

**TABLE 3:** Primary outcome (Length of stay) multivariate Cox regression model.

Variable factors	Beta	HR	95% CI		
			Lower limit	Upper limit	*p*-value
**Gender: Male**	−0.471	0.625	0.216	1.811	0.386
**Age: ≥ 60 years**	0.121	1.129	0.425	2.998	0.808
BMI > 25 kg/m^2^	0.919	2.507	0.743	8.459	0.138
**CCI Category: ≥ 1**	−0.776	0.460	0.168	1.259	0.131
**Vaccination: 3 doses completed**	−0.382	0.682	0.254	1.829	0.447
**Typing**	−0.116	0.891	0.479	1.656	0.714
**Nirmatrelvir/ritonavir initiate time (≤ 5 d)**	1.344	3.835	1.330	11.062	0.013

### 3.3 Secondary outcome: the time taken to reach Ct value ≥35

#### 3.3.1 Univariate analysis

Univariate Cox regression analysis showed that CCI ≥1 (HR 0.440, 95% CI 0.200–0.968, *p* = 0.042); BMI >25 kg/m^2^ (Kawaoka et al., 2022) (HR 4.772, 95% CI 1.782–12.78, *p* = 0.002), Nirmatrelvir/Ritonavir initiation time ≤5 days (HR 3.573, 95% CI 1.518–8.407, *p* = 0 .004) (see Table 4).

**TABLE 4:** Univariate Cox regression analysis of time to nucleic acid conversion for variables complying with PH hypothesis test.

Variable factors	HR	95% CI		
		Lower limit (days)	Upper limit (days)	*p*-value
**Gender: Male**	0.521	0.239	1.137	0.101
**Age: ≥ 60 years**	0.815	0.379	1.753	0.601
BMI > 25 kg/m^2^	4.772	1.782	12.78	0.002
**Underlying disease: Yes**	0.619	0.295	1.298	0.204
**Comorbidities: Yes**	0.707	0.319	1.565	0.392
**CCI Category: ≥ 1**	0.440	0.200	0.968	0.042
**Vaccination: 3 doses completed**	0.827	0.401	1.704	0.606
**Clinical classification**	0.962	0.584	1.583	0.877
**Admission to ICU: Yes**	0.303	0.089	1.035	0.057
**Laboratory tests on admission**				
Lymphocyte count: decreased	0.856	0.412	1.779	0.677
IL-6: Increased	1.641	0.710	3.796	0.247
CRP: Increased	1.142	0.546	2.389	0.724
**Nirmatrelvir/ritonavir initiate time (≤ 5 d)**	3.573	1.518	8.407	0.004
**Ct value before administration**				
ORF	0.999	0.942	1.059	0.969
N	0.984	0.925	1.047	0.609
**Time to resolution (days)**	0.984	0.928	1.045	0.605

**FIGURE 2:**
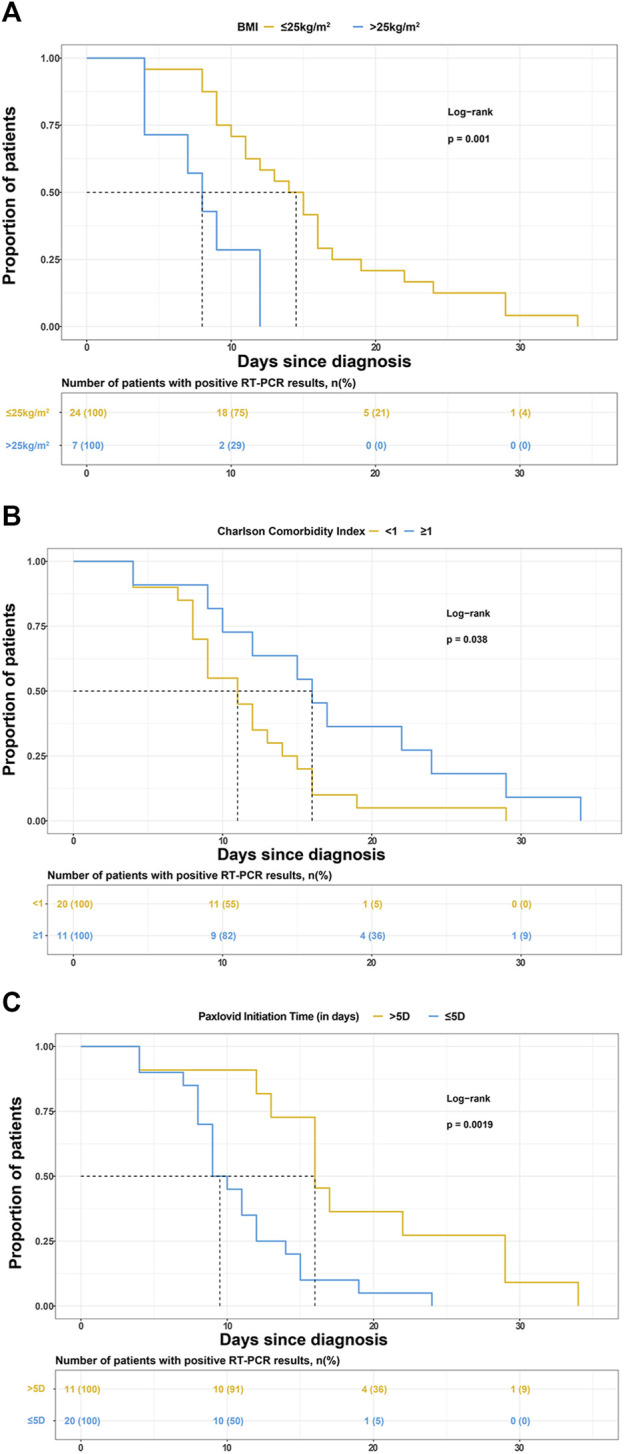
Kaplan-Meier curves for the proportion of patients with positive reverse transcription-polymerase chain reaction (RT-PCR) results for SARS-COV-2 infection in different groups. The median (interquartile range) time for the positive RT-PCR results converted to negative was **(A)** 14.5 days (9.5–18 days) in patients with BMI ≤25 kg/m2 and 8.5 days (4–12 days) in patients with BMI>25 kg/m2, **(B)** 11 days (8–14.5 days) in patients with Charlson comorbidity index (CCI) < 1 and 16 days (10–24 days) in patients with CCI>1, **(C)** 9.5 days (8–13 days) in patients treated with Paxlovid within 5 days since diagnosis and 16 days (13–29 days) in patients treated with Paxlovid beyond 5 days since diagnosis.

BMI, CCI category, and Nirmatrelvir/Ritonavir initiation time were selected to draw the K-M survival curve, as shown in Figures 2A–C.

#### 3.3.2 Multivariate Cox regression model analysis

We analyzed basic information (gender, BMI, age ≥60 years), underlying disease information (CCI ≥1), treatment and prevention (Nirmatrelvir/Ritonavir initiation time, completion of 3 vaccinations), and disease condition (severity classification). Among these variables, BMI >25 kg/m^2^ (HR 4.582, 95% CI 1.356–15.486, *p* = 0.014), and Nirmatrelvir/Ritonavir initiation time ≤ 5 d (HR 2.755, 95% CI 1.033–7.345, *p* = 0.043) were influencing factors, shown in Table 5. It was shown that BMI > 25 kg/m^2^ and Nirmatrelfir/ritonavir initiation time ≤5 days shortened time to Ct value ≥35 but CCI ≥1 prolonged time to Ct value ≥35.

**TABLE 5:** Multivariate Cox regression model for the secondary outcome (time to nucleic acid conversion).

Variable factors	Beta	HR	95% CI		
			Lower limit (days)	Upper limit (days)	*p*-value
**Gender: Male**	−0.571	0.565	0.188	1.698	0.309
**Age: ≥ 60 years**	−0.278	1.269	0.291	2.134	0.639
BMI > 25 kg/m^2^	1.522	4.582	1.356	15.486	0.014
**CCI Category: ≥ 1**	−1.069	0.343	0.119	0.993	0.049
**Vaccination: 3 doses completed**	−0.834	0.434	0.158	1.192	0.105
**Typing**	0.192	1.211	0.623	2.356	0.573
**Nirmatrelvir/ritonavir initiate time (≤ 5 d)**	1.013	2.755	1.033	7.345	0.043

## 4 Discussion

In this study, we found that initiation of treatment with Nirmatrelvir/Ritonavir within 5 days after the diagnosis of micron BA.2 variant of COVID-19 infection significantly shortened the length of hospital stay and the time for reaching Ct value ≥35, and these findings are consistent with the EPIC-HR (Evaluation of Protease Inhibition for COVID-19 in High-Risk Patients) study results. There was no death associated with COVID-19 in all patients who were administered Nirmatrelvir/Ritonavir. We also found that the Charlson comorbidity index (CCI) and BMI of patients were important factors affecting the time taken to reach Ct value ≥35.

In this study, all patients were infected with Omicron BA.2 variant of COVID-19 We found that timely treatment with Nirmatrelvir/Ritonavir helped inpatients shorten hospitalization stay and reduced their viral load (based on time taken to reach Ct value ≥35). This confirmed the effectiveness of Nirmatrelvir/Ritonavir in treating the Omicron BA.2 variant of COVID-19. These findings are similar to the study results of Najar-Debbinyand Sun et al. (Najjar-Debbiny et al., 2022; Sun et al., 2022) This also confirmed that 3CLpro inhibitors were effective against SARS-CoV-2mutant variants.

We found that that age, classification, and CCI ≥1 factors were not statistically significant in relation to the length of hospital stay. This is contrary to several published studies which indicate that older patients with COVID-19 are at higher risk and more prone to a prolonged hospital stay (da Costa Sousa et al., 2022; Xu et al., 2022; Zheng et al., 2020). In our opinion, one probable reason for the difference may be related to the small sample size of the present study. Meanwhile, more aggressive use of Paxlovid antiviral therapy in patients with high-risk factors, including advanced age and having high risk, may also lead to selection bias and thus outcome differences. This is similar to the study by Sun et al. which noted that immunosuppressed patients were treated with Nirmatrelvir/Ritonavir (da Costa Sousa et al., 2022).

CCI is a comorbidity scoring system used to measure comorbidities based on the severity of disease. The study by Sun et al. showed that the time taken to reach Ct value ≥35 was longer in patients with neurological or cardiovascular disease comorbidities and immunocompromised patients (Sun et al., 2022). In this study, the secondary outcome (the time taken to reach Ct value ≥35) suggests that patients with CCI ≥1 can prolong the time of Ct value ≥35.

We also found in this study that BMI >25 kg/m^2^ shortened the time required to achieve a Ct value ≥35 in patients, and these results are different from that of Mufarrih et al. (2022). This difference may be explained by the different CCI in patients with BMI ≤25 kg/m^2^ and BMI >25 kg/m^2^. In our study, we found that CCI ≥1 was a factor affecting the time taken to reach Ct value ≥35, and seven patients (29.2%) with BMI ≤25 kg/m^2^ had a CCI ≥3, while patients with BMI >25 kg/m^2^ did not have CCI ≥3. The relationship between BMI and the time taken to reach Ct value ≥35 remains to be explored in large samples in future studies.

There are several limitations in this study: First, this is a retrospective study with a small sample size, and this can cause sample selection bias. Second, according to the “Novel Coronavirus Pneumonia Treatment Protocol (Trial Version 9)" and the instructions for use of Paxlovid in China, the indication for the use of Paxlovid is “for adults with mild to moderate COVID-19 with high-risk factors for progression to severe disease as soon as possible within 5 days of diagnosis”. However, in this study, some patients with infections older than 5 days and patients with severe disease were also treated with Paxlovid. Third, this study lacks a control group of COVID-19 inpatients who did not receive NiR, which is not conducive to the comparison of results.

## 5 Conclusion

In conclusion, in this study, we found that during the Omicron BA.2 epidemic, administering Nirmatrelvir/Ritonavir early (≤5 days) was highly effective in shortening the length of hospital stay and faster clearance of viral load (measured in terms of the time taken to reach Ct value ≥35).

## Data Availability

The original contributions presented in the study are included in the article/supplementary material, further inquiries can be directed to the corresponding author.
